# Retrieval practice as a learning strategy for individuals with Down syndrome A preliminary study

**DOI:** 10.1590/1980-57642018dn13-010012

**Published:** 2019

**Authors:** Daniela Siqueira Veloso Starling, Bruna Fernanda Tolentino Moreira, Antônio Jaeger

**Affiliations:** 1 PhD in Neuroscience, Federal University of Minas Gerais, MG, Brazil.; 2 Graduate student in Psychology, Federal University of Minas Gerais, MG, Brazil.; 3 PhD in Psychology, Federal University of Rio Grande do Sul, RS, Brazil.

**Keywords:** Down syndrome, retrieval-practice, testing-effect, learning, síndrome de Down, prática de recuperação, efeito de teste, aprendizagem

## Abstract

Remembering recently studied materials (i.e., retrieval practice) is more beneficial for learning than restudying these materials.

**Objective:**

To investigate whether retrieval practice benefits learning for individuals with Down syndrome.

**Methods:**

Eighteen individuals with Down syndrome (mean age=21.61 years, *SD*=5.93) performed a task entailing a first read of an encyclopedic text covering a series of target words. After reading the text twice, participants recalled half of the target words (retrieval practice), and reread the other half (restudy). After 48 hours, participants answered a multiple-choice test including all target words. Subsequently, WASI’s Vocabulary and Matrix reasoning subtests were administered to estimate intelligence.

**Results:**

The benefit of retrieval practice for learning was numerically greater than the benefit of restudy, although this advantage did not reach statistical significance. Inspection of individual data suggested that the benefit of retrieval practice was greater than the benefit of restudy for the majority of the participants, independently of the participants’ vocabulary or reasoning abilities.

**Conclusion:**

Although more research is needed before retrieval practice can be recommended as a learning strategy for individuals with Down syndrome, the data suggest that retrieval practice can be a useful teaching tool for at least part of this population.

## RESUMO.

Lembrar materiais recentemente estudados (i.e., prática da recuperação) é melhor para a aprendizagem do que reestudar esses materiais.

**Objetivo:**

Investigar se a prática da recuperação beneficia a aprendizagem de indivíduos com síndrome de Down.

**Métodos:**

Dezoito indivíduos com síndrome de Down (idade média=21,61, DP=5,93) leram um texto enciclopédico com várias palavras-alvo. Depois de o texto ser lido duas vezes, metade das palavras-alvo (prática de recuperação) foi relembrada, e metade foi relida (reestudo). Após 48 horas, os participantes responderam a um teste de múltipla escolha incluindo todas as palavras-alvo. A inteligência dos participantes foi avaliada pelos subtestes Raciocínio matricial e Vocabulário do WASI.

**Resultados:**

O benefício da prática de recuperação para o aprendizado foi melhor quando comparado ao reestudo, embora essa diferença não tenha alcançado significância estatística. A inspeção de dados individuais sugeriu que o benefício da prática de recuperação foi melhor que o benefício do reestudo para a maioria dos participantes, independentemente do vocabulário ou capacidade de raciocínio.

**Conclusão:**

Embora mais pesquisas sejam necessárias para recomendar o uso dessa estratégia de aprendizado para essa população, os dados sugerem que a prática de recuperação pode ser uma ferramenta de ensino útil, para pelo menos parte dessa população.

The practice of remembering studied materials (i.e., retrieval practice) promotes greater subsequent learning of those materials than repeatedly studying them (i.e., restudy). This mnemonic phenomenon, called the “testing-effect”, has been intensely studied in the last decade^1^^-^ 5 and shown to be a promising teaching strategy to improve learning in classroom settings.^6^

The benefits of retrieval practice for education have been investigated in students from elementary to graduate school.^7^^-^ 14 Studies conducted with elementary school children demonstrated that several types of memory tests improved long-term learning of studied materials.^7^^,^ 8 ^,^ 10 ^,^ 12 ^,^ 13 However, despite the large body of evidence suggesting that retrieval practice is beneficial for long-term learning, only a few studies have examined whether it interacts with differences among individuals regarding intelligence or specific cognitive abilities. Thus, to date, few studies have investigated whether retrieval practice can be a beneficial learning strategy for individuals with diverse cognitive and developmental characteristics.

The few studies investigating this issue have shown that retrieval practice is more beneficial than restudy for both younger and older individuals,^15^ individuals with neurological conditions,^16^^,^ 17 those with low general fluid intelligence and low episodic memory,^18^ individuals with higher working memory capacities,^19^ and for children with a wide range of reading skills.^20^^,^ 21 In contrast, retrieval practice is apparently no more beneficial than restudy for individuals with attention-deficit hyperactivity disorder.^22^

Previous research has revealed mixed results concerning the association of intelligence with the efficacy of retrieval practice for learning. While Brewer and Unsworth^18^ showed that retrieval practice is more beneficial for low- than for high-general fluid intelligence young adults, other authors^20^ found no associations between intelligence and the testing-effect in third grade children. Thus, the current knowledge concerning the interaction of retrieval practice with intelligence remains embryonic. Unfortunately, the picture is even more incipient regarding the knowledge about the potential benefits of retrieval practice for individuals with intellectual disabilities, such as those with Down Syndrome (DS), since to date no studies have addressed this issue. This is especially important considering the educational demands specific to this population.^23^

For this reason, in the current study we examined whether individuals with Down syndrome could be benefited by retrieval practice. To accomplish this, these individuals were asked to perform fill-in-the-gap tests on a narrative text about the Sun.^20^ In a multiple-choice test conducted after an interval of 48 hours, the participants’ long-term memory for the fill-in-the-gap items was compared with their memory for reread items (i.e., words from the text that were not missing during the fill-in-the-gap test). Because no association between the benefits of retrieval practice and intelligence was found in prior studies,^20^ we expected to find evidence showing that this strategy can improve learning for these individuals.

## METHODS

### Participants

Twenty-five participants were initially recruited for this study. However, four participants were subsequently excluded due to absence at the second session of the experiment, and three were excluded by request of their families to withdraw from the study. Thus, the final sample comprised eighteen teenagers and adults with Down syndrome. Participant age ranged from 12 to 37 years (mean=21.61, *SD*=5.93, 11 females) and Down syndrome was identified in these individuals through phenotype inspection. All participants lived with their families in middle class neighborhoods in the city of Belo Horizonte, in the southeastern state of Minas Gerais, Brazil. All subjects were literate (i.e., able to read or write without assistance), and had been enrolled at regular or special schools for at least 5 years. Parents were informed of the study and a written consent form was obtained for each participant according to the Institutional Review Board of the Federal University of Minas Gerais (UFMG)*.* The Research Ethics Committee approved the study under Approval Number 39898514.1.0000.5149.

### Materials and design

**Figure 1:**
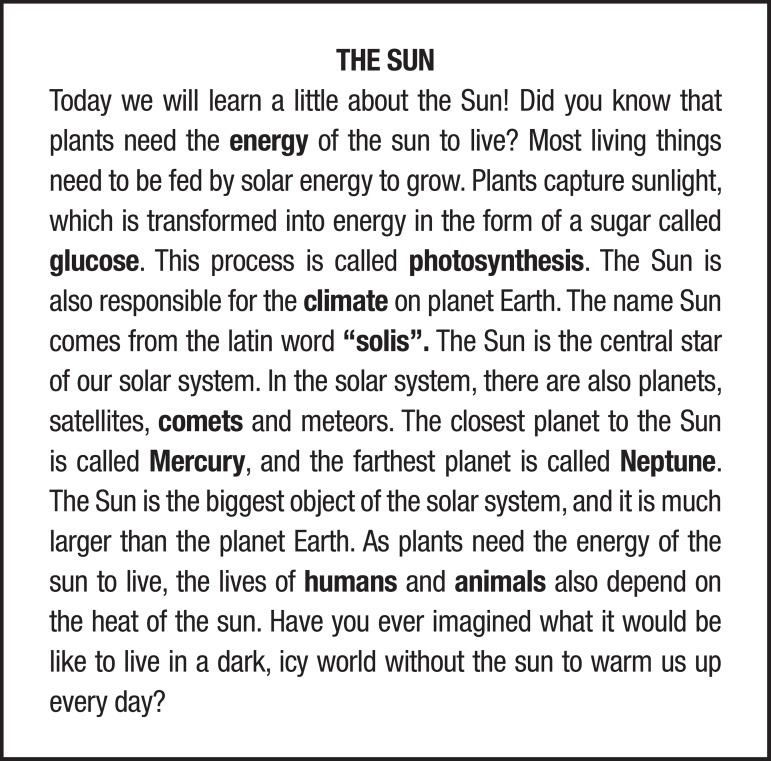
Text entitled “The Sun” used to test retrieval practice. The target-words are in bold.

Two instruments were used in the current study. The Wechsler Abbreviated Intelligence Scale (WASI), to estimate the participants’ intelligence, and an encyclopedic text about the Sun, to investigate retrieval practice. The WASI is a brief test for assessing intelligence, applicable to individuals aged 6 to 89 years. The subtests Vocabulary and Matrix reasoning were used to estimate participant IQ. The retrieval practice material consisted of a 175-word text about the Sun (see Figure 1), adapted from a 335-word text used in a prior retrieval practice study with third grade children,^20^ and from a text used in an earlier study on the testing-effect.^1^ The text was analyzed by directors, teachers and coordinators experienced in working with individuals with Down syndrome, and was considered appropriate for this population. Ten words from the text were selected as “target-words”, which were then assigned for the retrieval practice or for the restudy condition. The full text, along with the target words (in bold), can be seen in Figure 1. The assignment of the target-words for each experimental condition was counterbalanced among participants.

The activities promoting retrieval practice and restudy were organized in 4 booklets. Booklet 1 contained the complete 175-word text, and participants were instructed to read the whole text twice. After they had finished the second reading, participants performed a brief buffer task involving the solving of simple addition and subtraction operations. Booklet 2 contained the same text, but in place of 5 of the target-words, there was a gap with the corresponding target-word’s word stem (e.g., *so____* for the target-word *solis*). The remaining 5 target-words were presented in full, but were in bold font. The participants were instructed to reread the text while completing the 5 missing target-words from their word stems. After finishing this activity, participants performed another buffer task involving simple mathematical operations. Booklet 3 was similar to booklet 2, with the exception that no word stems were presented in place of the missing words. Participants were instructed to fill in the gaps with the corresponding target-word while rereading the whole text again.

Finally, booklet 4 contained the 10 multiple-choice questions, which were given to the participants 48 hours after completion of activities from booklet 3. The questions required the completion of 10 sentences extracted from the text, with their respective target-words presented along with 3 distractor alternatives. The distractor alternatives were similar to the target words and equally plausible for those with no previous knowledge about the subject (e.g., the word sun derives from the Latin word: (a) *solum*, (b) *solis*, (c) *solen*, (d) *soales*). Participants were instructed to choose the correct alternative according to the text about the Sun, read in booklets 1 to 3.

### Procedures

**Figure 2:**
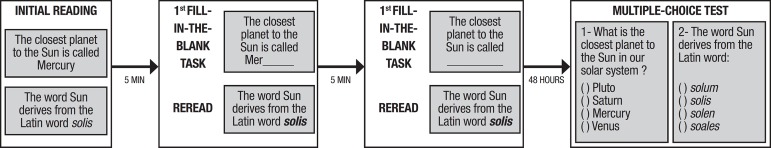
Schematic of experimental design. After the initial reading of the text, participants restudied and retrieved different target words from the text. After retrieval practice, participants performed a multiple-choice recognition task with both the studied and tested target words.

The experiment took place in two sessions separated by a 48-hour interval. In the first, participants received booklet 1 and read the text “The Sun” twice. After finishing the reading, they had five minutes to complete the buffer task. At this point, some participants asked whether, instead of solving the mathematical operations, they could draw. The experimenter allowed the change, observing that the goal of the buffer task was to keep participants from dwelling on the contents of the text and, thus, engagement in the buffer task was more important than its nature. After completing booklet 1, participants received booklet 2, in which they reread the text, performed the first fill-in-the-gaps task (with word stems) and completed another buffer task. Finally, in booklet 3, participants reread the text and performed the second fill-in-the-gaps task (without word stems) (Figure 2).

After an interval of 48 hours, participants received booklet 4, in which they answered ten multiple-choice questions involving the target words. Five of the questions comprised target words that were previously retrieved in the fill-in-the-gap tasks, and five comprised target words that had only been reread. After completing booklet 4, the Vocabulary and Matrix reasoning subtests of the WASI were administered. Both sessions were held individually at participant’s homes, and lasted approximately 30 minutes each.

### Data analysis

Initially, with the goal of estimating the participants’ intelligence, the estimated IQ from the WASI subtests was analyzed and the t-scores estimated from each subtest were then reported. After this, the goal was to examine participants’ basic memory performance, accomplished by analyzing the performances on the two fill-in-the-gap tests (i.e., with and without word stems). Statistical analysis was then conducted, with the goal of examining whether retrieval practice was more beneficial than restudy. This analysis consisted of a *t*-test comparing the final multiple-choice test performance for items from the retrieval practice condition with items from the restudy condition. In order to examine whether retrieval practice was beneficial for most participants, despite the absence or presence of statistical significance on the analysis of the whole group, the data regarding the magnitude of the testing effect (i.e., retrieval practice scores minus restudy scores) were reported for each participant.

Finally, to investigate whether intelligence was associated with memory performance and with the testing effect, correlation analyses were performed between intelligence scores and the memory tests (both fill-in-the-gaps tests) and between the intelligence scores and the testing effect. For all memory tests, one point was assigned for each correct response, and zero for each blank or incorrect response. Thus, the maximum score in each retrieval practice fill-in-the-gaps test was 5, and the maximum overall score on the final multiple-choice test was 10 (i.e., 5 points for the retrieval and 5 for the restudy conditions). For the correlation analysis, the magnitude of the testing effect was estimated by subtracting restudy scores from test scores (i.e., retrieval minus restudy). This measurement represented the mnemonic advantage or disadvantage of retrieval practice over restudy for each participant. For all statistical analysis, differences with *p* values ≤ 0.05 were considered significant.

## RESULTS

The average intelligence of the participants, as estimated by the WASI subtests was 56.33 (*SD*=7.33, range=40-65), corresponding to an average percentile of 0.2. Of the 18 participants included in the study, 5 presented moderate intellectual disability (estimated IQ 40-46) and 13 had mild intellectual disability (estimated IQ 50-65). The average Vocabulary and Matrix Reasoning *t*-scores were 21.67 (*SD=*8.85) and 9.17 (SD=5.45), respectively.

Considering memory performance, in the first fill-in-the-gaps test (with word stems), participants recalled an average of 3.22 (*SD*=1.59) out of 5 target words, whereas in the second fill-in-the-gaps test (without word stems), they recalled an average of 1.56 (*SD*=1.69). On the final multiple-choice recognition test administered two days later, participants recognized more target words from the retrieval practice condition than the restudy condition task. Participants recalled an average of 2.78 (*SD*=1.44) out of 5 target words for the retrieval practice condition, and an average of 2.39 (*SD*=1.72) out of 5 target words for the restudy condition, a difference, however, that did not reach statistical significance, *t*(17)=1.16, *p=*0.26.

**Figure 3:**
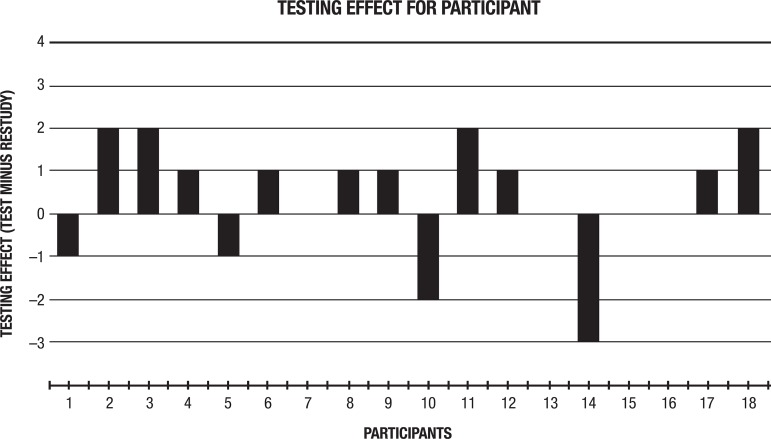
The magnitude of Testing Effect. This figure illustrates the magnitude of the testing effect (i.e., retrieval scores minus restudy scores) for each participant. Thus, it is possible to note that retrieval practice was more advantageous than restudy for most participants (10). The Testing-effect was equivalent to restudy for 4 participants and was more disadvantageous than restudy for only 4 participants.

Nevertheless, because the current sample of participants and number of target-words were relatively small, statistical comparisons may not have captured individual patterns, where these can be informative for future research or educational purposes. Thus, as can be seen in Figure 3, plots of the magnitude of the testing-effect (retrieval scores minus restudy scores) of each participant reveal that retrieval practice was more advantageous than restudy for most participants. Therefore, the testing effect proved more advantageous than restudy for 10 participants, was equivalent to restudy for 4 participants, and was more disadvantageous than restudy for 4 participants.

Memory performance was positively correlated with IQ in the retrieval practice tests (first fill-in-the-gaps test, *r*=0.63, *p*<0.01; second fill-in-the-gaps test, *r*=0.53, *p*=0.02), although it was not significantly correlated with performance in the delayed multiple-choice test (*r*=0.34, *p*=0.16). More importantly, however, no significant correlation between IQ and the magnitude of the testing effect was found (*r*= -0.08, *p*=0.75). Thus, although IQ was associated with memory performance, it was not associated with the magnitude of the testing effect, suggesting that retrieval practice may be equally beneficial for individuals with diverse intelligence scores.

## DISCUSSION

The current study shows that, for several individuals with Down syndrome, retrieval practice may be an effective alternative learning strategy, although this possibility was only observed when we assessed the magnitude of the testing effect using a more individualized approach. In other words, the statistical analysis did not show significant results here. Interestingly, although intelligence correlated with performance on the fill-in-the-gap tests, it did not correlate with the magnitude of the testing effect. This latter finding suggests that potential advantages promoted by retrieval for long-term retention are not affected by intelligence, thus indicating that individuals with diverse intelligence scores can benefit from this strategy. These issues are discussed further below.

The lack of statistically significant results for the testing effect in the overall group analysis may appear discouraging at first. A reason for this unexpected result might be related to the size of the current sample, or to the small number of target-words used. These are important limitations of the current study. Perhaps, with a larger sample and more target-items, the favorable numerical difference for retrieval versus restudy might reach statistical significance, and future research will be necessary to verify this possibility. On the other hand, it is possible that retrieval practice is not really advantageous for all Down syndrome individuals. Perhaps, because of individual differences not assessed in the current study (other than IQ), some individuals with Down syndrome are more benefited by repeatedly studying the materials to be learned than recalling them. As mentioned in the Introduction section, research concerning the interaction between the testing effect and individual differences is still in its infancy^18^^,^ 19 ^,^ 21 ^,^ 22 and, with few exceptions, research in this field has neglected individual performances.

Regarding the application of retrieval practice in educational settings, a more personalized approached might be useful. More specifically, it might be important to assess whether retrieval practice is a beneficial learning strategy for each student. For example, in the current study, the strategy is possibly not advantageous for participants 1, 6, 11, and 15 (see Figure 3). For students showing these patterns, instead of retrieval practice, alternative strategies could be employed in actual educational settings.

However, the lack of statistical significance for the testing effect in the current study might be related to the accuracy rate on the first and second fill-in-the-gap tests. For example, on the first test (with word stems), participants recalled an average of 64.4% of the target-words, while on the second (without word stem) they recalled an average of 31.2% of the target-words. This finding is consistent with previous research exploring the difficulties encountered by individuals with Down syndrome on long-term memory tests.^24^^-^ 26 Thus, this overall memory limitation may be the main cause of the lack of positive testing effects found in the current study, since to promote testing-effects, it is important that most materials are recalled during the practice of retrieval.^21^

Alternatively, the current choice of materials might have influenced the results. Although the text used in the experiment was previously analyzed by professionals involved in the education of individuals with intellectual disabilities, some of the participants might have found them difficult. This issue is critical, especially considering that Down syndrome individuals typically exhibit more difficulties with verbal materials, relative to visual materials.^27^ Future research should take this issue into consideration, and explore whether retrieval practice can be more consistently beneficial when visual materials are used.

Another limitation of the current study is that the identification of Down syndrome in the current sample was performed only through phenotype inspection. Because cognitive performance may be influenced by the type of chromosome abnormality exhibited by the individual, future research should examine this issue more closely by perhaps recruiting participants who were diagnosed based on karyotype tests. This is especially important considering that individual differences may play an important role in the effectiveness of the testing effect.

Finally, considering the correlations between IQ and memory, as assessed by the two fill-in-the-gap tests, the results showed that memory was associated with IQ. Importantly, however, IQ was not associated with the magnitude of the testing-effect. Interestingly, in an earlier study using similar materials^20^, but employing the Raven Coloured Progressive Matrices^28^ to assess intelligence in typically developing children, no correlation between IQ and the magnitude of the testing effect was found.

Overall, the convergence between current and previous data^20^ suggests that retrieval practice can be beneficial for individuals with a wide range of IQ scores. However, this benefit is not evident for all individuals. Thus, future research should elucidate which individual characteristics are associated with advantages in using retrieval over restudy.

## References

[1] Roediger HL, Karpicke JD (2006). Test-enhanced learning:Taking memory tests improves long-term retention. Psychol Sci.

[2] Karpicke JD, Roediger HL (2007). Repeated retrieval during learning is the key to long-term retention. J Mem Lang.

[3] Butler AC, Roediger HL (2008). Feedback enhances the positive effects and reduces the negative effects of multiple-choice testing. Mem Cogn.

[4] Halamish V, Bjork RA (2011). When does testing enhance retention? A distribution-based interpretation of retrieval as a memory modifier. J Exp Psych Learn Mem Cogn.

[5] Eisenkraemer RE, Jaeger A, Stein LM (2013). A systematic review of the testing effect in learning. Paidéia.

[6] Adesope OO, Trevisan DA, Sundararajan N (2013). Rethinking the use of tests:A meta-analysis of practice testing. Rev Educ Res.

[7] Carpenter SK, Pashler H, Cepeda NJ (2009). Using tests to enhance 8th grade students' retention of U.S. history facts. Appl Cog Psychol.

[8] McDaniel MA, Agarwal PK, Huelser BJ, McDermott KB, Roediger HL (2011). Test-enhanced learning in a middle school science classroom:The effects of quiz frequency and placement. J Educ Psychol.

[9] Ramrajee SN, Sable PL (2011). Comparison of the effect of post-instruction multiple-choice and short-answer tests on delayed retention learning. Australas Med J.

[10] Roediger HL, Agarwal PK, McDaniel MA, McDermott KB (2011). Test-enhanced learning in the classroom:long-term improvements from quizzing. J Exp Psychol Appl.

[11] McDaniel MA, Wildman KM, Anderson JL (2012). Using quizzes to enhance summative-assessment performance in a web-based class: An experimental study. J Applied Res Mem Cogn.

[12] McDaniel MA, Thomas RC, Agarwal PK, McDermott KB, Roediger HL (2013). Quizzing in middle-school science: Successful transfer performance on classroom exams. Applied Cogn Psychol.

[13] Lipko-Speed A, Dunlosky J, Rawson KA (2014). Does testing with feedback help grade-school children learn key concepts in science?. J Appl Res Mem and Cogn.

[14] Lipowski SL, Pyc MA, Dunlosky J, Rawson KA (2014). Establishing and explaining the testing effect in free recall for young children. Develop Psychol.

[15] Tse CS, Balota DA, Roediger HLIII (2010). The benefits and costs of repeated testing on the learning of face-name pairs in healthy older adults. Psychol Aging.

[16] Sumowski JF, Chiaravalloti N, DeLuca J (2010). Retrieval practice improves memory in multiple sclerosis. Clin Appl Test Effec Neuropsychol.

[17] Sumowski J, Wood H, Chiaravalloti N, Wylie G, Lengenfelder J, Deluca J (2010). Retrieval Practice: A simple strategy for improving memory after traumatic brain injury. J Int Neuropsychol Soc.

[18] Brewer GA, Unsworth N (2012). Individual differences in the effects of retrieval from long-term memory. J Mem and Lang.

[19] Tse CS, Pu X (2012). The effectiveness of test-enhanced learning depends on trait test anxiety and working-memory capacity. J Exp Psychol Appl.

[20] Jaeger A, Eisenkraemer RE, Stein LM (2015). Test-enhanced learning in third-grade children. Educ Psychol.

[21] Karpicke JD, Blunt JR, Smith MA (2016). Retrieval-based learning:positive effects of retrieval practice in elementary school children. Front Psychol.

[22] Dudukovic NM, Gottshall JL, Cavanaugh PA, Moody CT (2015). Diminished testing benefits in young adults with attention-deficit hyperactivity disorder. Memory.

[23] Fidler DJ, Nadel L (2007). Education and children with Down syndrome: Neuroscience, development, and intervention. Ment Retard Dev Dis Res Rev.

[24] Carlesimo GA, Marotta L, Vicari S (1997). Long-term memory in mental retardation:evidence for a specific impairment in subjects with Down's syndrome. Neuropsychologia.

[25] Jarrold C, Baddeley AD, Phillips C (2007). Long-term memory for verbal and visual information in Down syndrome and Williams syndrome: performance on the doors and people test. Cortex.

[26] Costanzo F, Varuzza C, Menghini D, Addona F, Gianesini T, Vicari S (2013). Executive functions in intellectual disabilities:a comparison between Williams syndrome and Down syndrome. Res Dev Disabil.

[27] Lanfranchi S, Cornoldi C, Vianello R (2004). Verbal and visuospatial working memory deficits in children with Down syndrome. Am J Ment Retard.

[28] Angelini AL, Alves IC, Custódio EM, Duarte WF, Duarte JL (1999). Matrizes progressivas coloridas de Raven: escala especial.

